# Supplementary material for: The impact of mating and sugar feeding on blood-feeding physiology and behavior in the arbovirus vector mosquito Aedes aegypti
Source: PLoS Negl Trop Dis. 2021 Sep 30;15(9):e0009815. doi: 10.1371/journal.pntd.0009815 (PMC8509887; doi:10.1371/journal.pntd.0009815)
Supplement: S1 Text — (DOCX) [file pntd.0009815.s001.docx]

**Supporting information:**

**Results**

To test whether virgin and mated field-caught females ingested similar amounts of blood, we improvised a field-appropriate method for assessing degree of abdominal distension that used femur widths to measure the inter-sclerite distances (ISDs) of abdomens. Abdominal distension varied significantly by mating and blood engorgement level (GZLM: treatment, p<0.0001; S7 Fig A). As anticipated, there was a significant correlation between ISDs and the proportion of a female’s abdomen that was filled with blood (H-B post-hoc tests), suggesting that ISDs are a useful proxy for gauging abdominal distension resulting from blood meal engorgement and subsequent digestion in field settings where other methods are impractical (S2 Table). Surprisingly, however, we found that mated females displayed wider abdomens than virgins (H-B post-hoc tests), a pattern that occurred even shortly after feeding in the abdomens of females with fresh, red blood (independent samples T-test: p=0.008; S7 Fig B).

As expected, nearly all mated females we examined had developed vitellogenic-stage eggs due to blood feeding prior to their most recent meal (Fig 6B). We therefore examined the extent to which the differences in virgin and mated female abdomen size may have resulted from abdominal distention due to egg development and laying as opposed to larger blood meals. When analyzing virgin and mated ISDs based on whether females contained pre-vitellogenic or vitellogenic stage eggs, we found a significant mating and oogenesis effect (GZLM: treatment, p<0.0001; S7 Fig C), with virgin and mated female ISDs differing amongst females with pre-vitellogenic stage eggs, but not those that contained vitellogenic stage eggs (H-B post-hoc tests). Although we cannot rule out potential effects arising from differences in genetic backgrounds between our laboratory (Thai) and field (Colombian) mosquitoes, our findings in field-collected mosquitoes are consistent with the idea that mated females have greater blood meal engorgement than virgins due to effects on abdominal distension resulting from egg development and egg laying.

To further examine whether mated females display greater abdominal distension compared to virgins due to factors related to egg development in addition to blood meal engorgement, we measured the ISDs of females from our controlled laboratory blood meal digestion experiments (Fig 2) since these females were nulliparous prior to blood feeding (S7 Fig D). As expected, ISDs in this controlled experiment declined steadily over time due to diuresis and blood digestion (GZLM: time point, p<0.0001). ISD’s also varied significantly by treatment (GZLM: treatment, p<0.0001; trial, p=0.402), but this effect stemmed from largely a single mated group (sugar mated) rather than from consistent mating, MAG, or sugar feeding effects (H-B post-hoc tests). When laboratory specimen ISDs were analyzed by either the proportion of the abdomen that was filled with blood (GZLM: treatment, p=0.002; time point, p<0.0001; trial, P=0.368) or by the proportion of abdomens that contained fresh blood (GZLM: treatment, p=0.006; time point, p<0.0001; trial, p=0.028) we again observed significant differences in abdominal distension over time and between treatments (S8 Fig). We observed a consistent, albeit statistically non-significant trend for greater distension in mated and MAG-injected females compared to non-injected virgins and saline-injected virgins, respectively. However, statistically significant differences in ISD between treatments occurred only occasionally due to sugar feeding, injection treatment, or the proportion of a female’s abdomen that was filled with blood (H-B post-hoc tests). Together, our results indicate that under controlled conditions where egg development and other factors are accounted for, mating and MAG do not lead to increased blood meal engorgement in an initial blood meal. However, additional work is needed to understand their effects in the field.
