# Supplementary figures and images for: The impact of mating and sugar feeding on blood-feeding physiology and behavior in the arbovirus vector mosquito Aedes aegypti
Source: PLoS Negl Trop Dis. 2021 Sep 30;15(9):e0009815. doi: 10.1371/journal.pntd.0009815 (PMC8509887; doi:10.1371/journal.pntd.0009815)

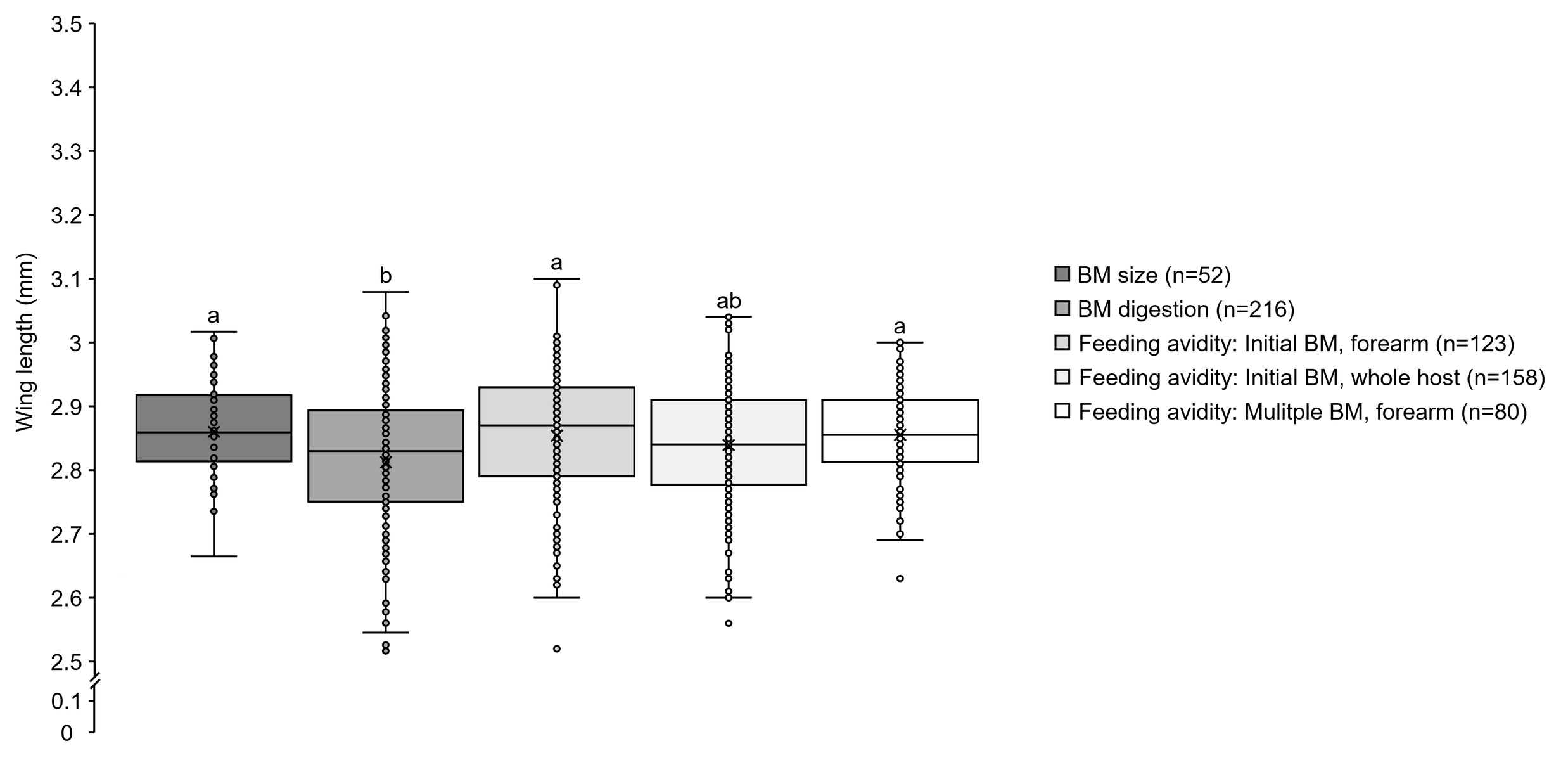

Supplement: S1 Fig — Despite a statistically significant experiment effect (GZLM: p = 0.001), post-hoc analysis showed only minor variation in wing lengths in the blood meal digestion experiments compared to the other experiments. Whiskers denote the minimum and maximum values. Box plots display the boundaries of the first (bottom) and third (top) quartiles, median lines, mean markers (“x”), and individual data points, including outliers. Letters above box and whisker plots denote H-B-corrected post-hoc comparison p-values. (TIF) [file pntd.0009815.s002.tif]

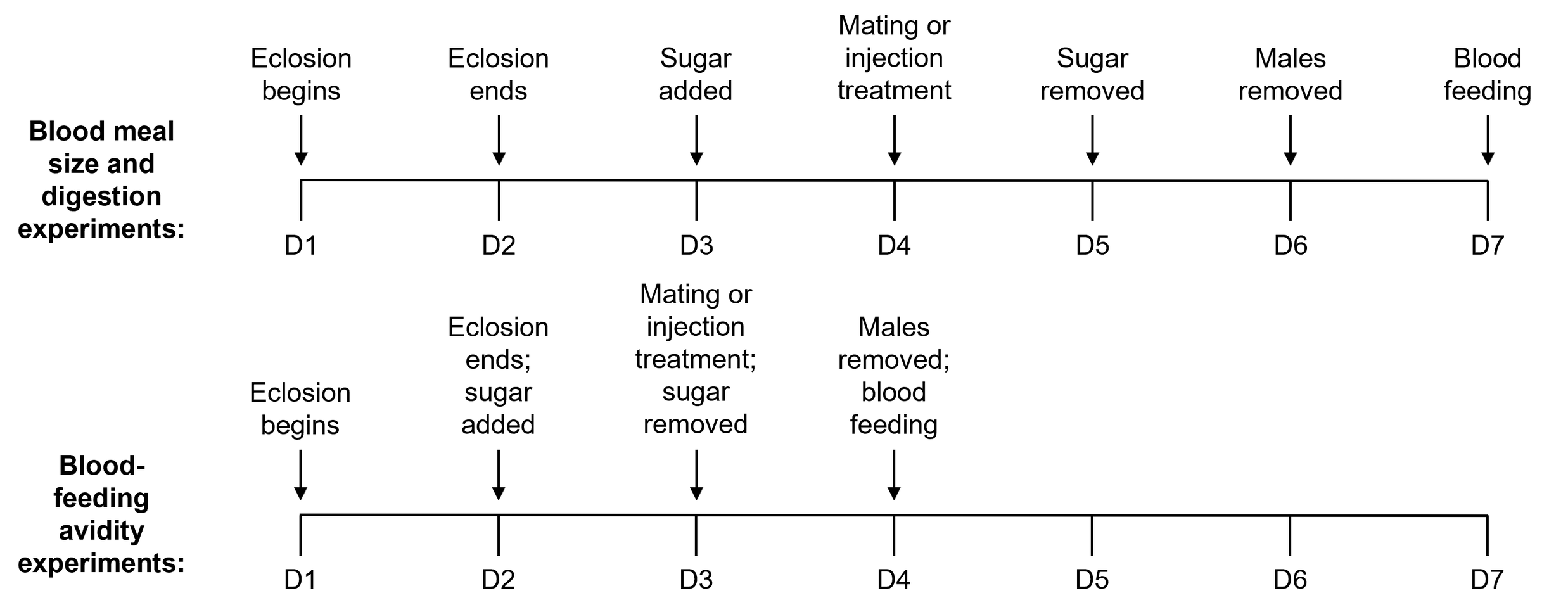

Supplement: S2 Fig — Abbreviation: D, day. (TIF) [file pntd.0009815.s003.tif]

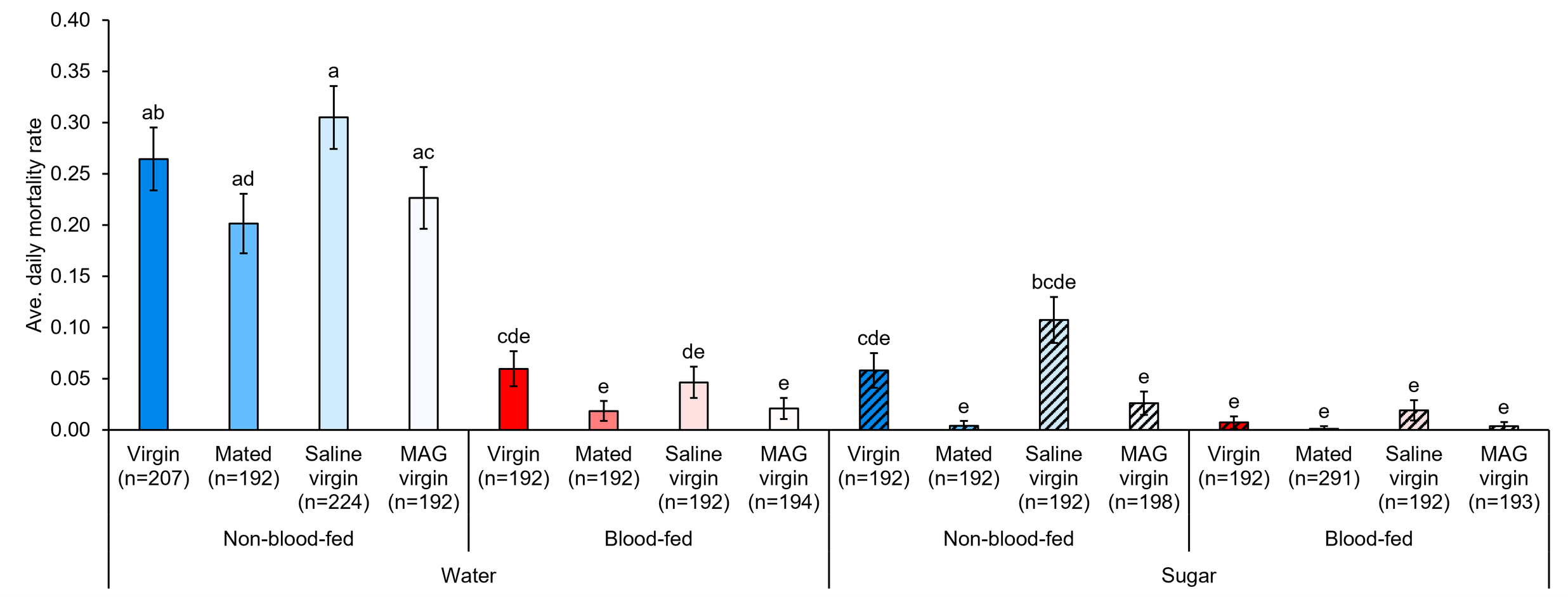

Supplement: S3 Fig — These results confirm earlier reports of increased survival due to mating [37,40], MAG injection [40], as well as blood and sugar feeding [94]. Error bars denote SE. Letters above columns denote H-B-corrected post-hoc comparison p-values. (TIF) [file pntd.0009815.s004.tif]

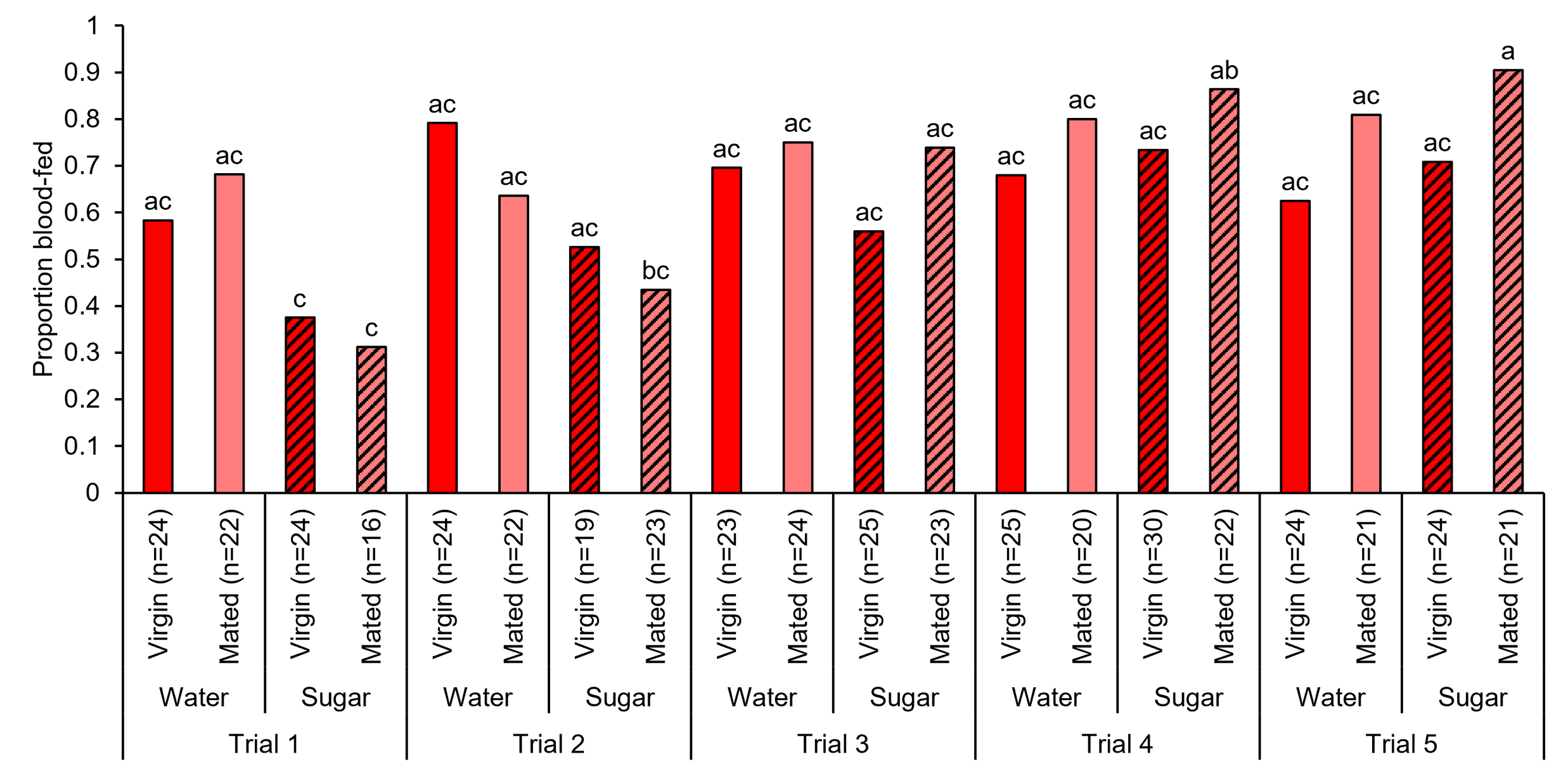

Supplement: S4 Fig — Analyzing combined mating treatment and sugar feeding treatment groups separately by trial showed that sugar-fed, but not water-fed female feeding avidity increased over the course of our experimental trials. Letters above columns denote H-B-corrected post-hoc comparison p-values. (TIF) [file pntd.0009815.s005.tif]

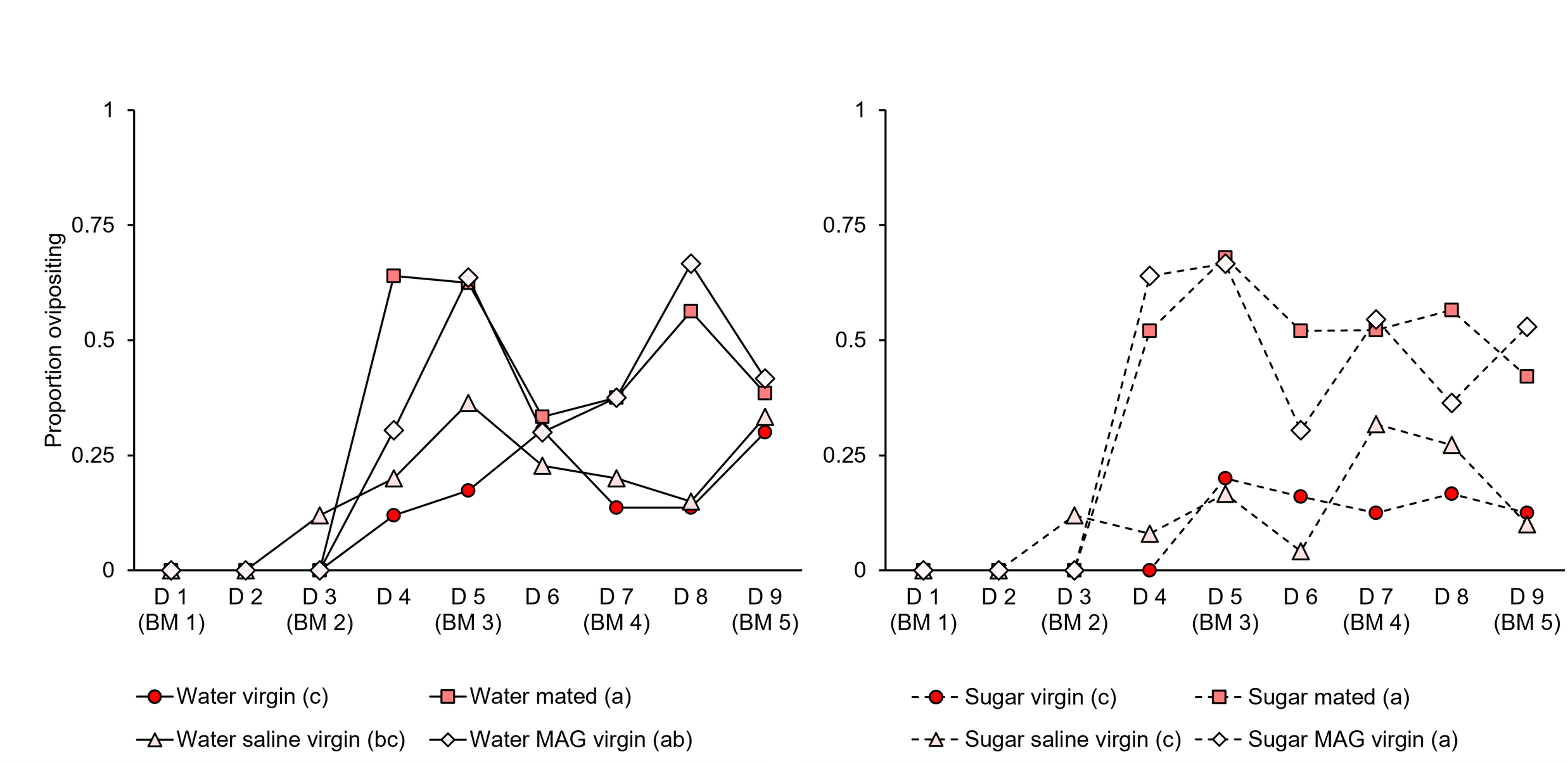

Supplement: S5 Fig — Detailed egg laying data collected from trial three of the multiple blood meal feeding avidity experiments (n = 75 total females per group) showed that combined mating treatment and sugar feeding treatment (GZLM: treatment, p<0.0001) and gonotrophic cycle day (day, p<0.0001) significantly affected oviposition behavior. Similar trends were observed, but not quantified, for trials one and two. For comparison of egg clutch one and two egg laying peaks with feeding avidity data, see Fig 5. Letters in parentheses next to the treatment group names denote H-B-corrected post-hoc comparison p-values. Abbreviations: D, day; BM, blood meal. (TIF) [file pntd.0009815.s006.tif]

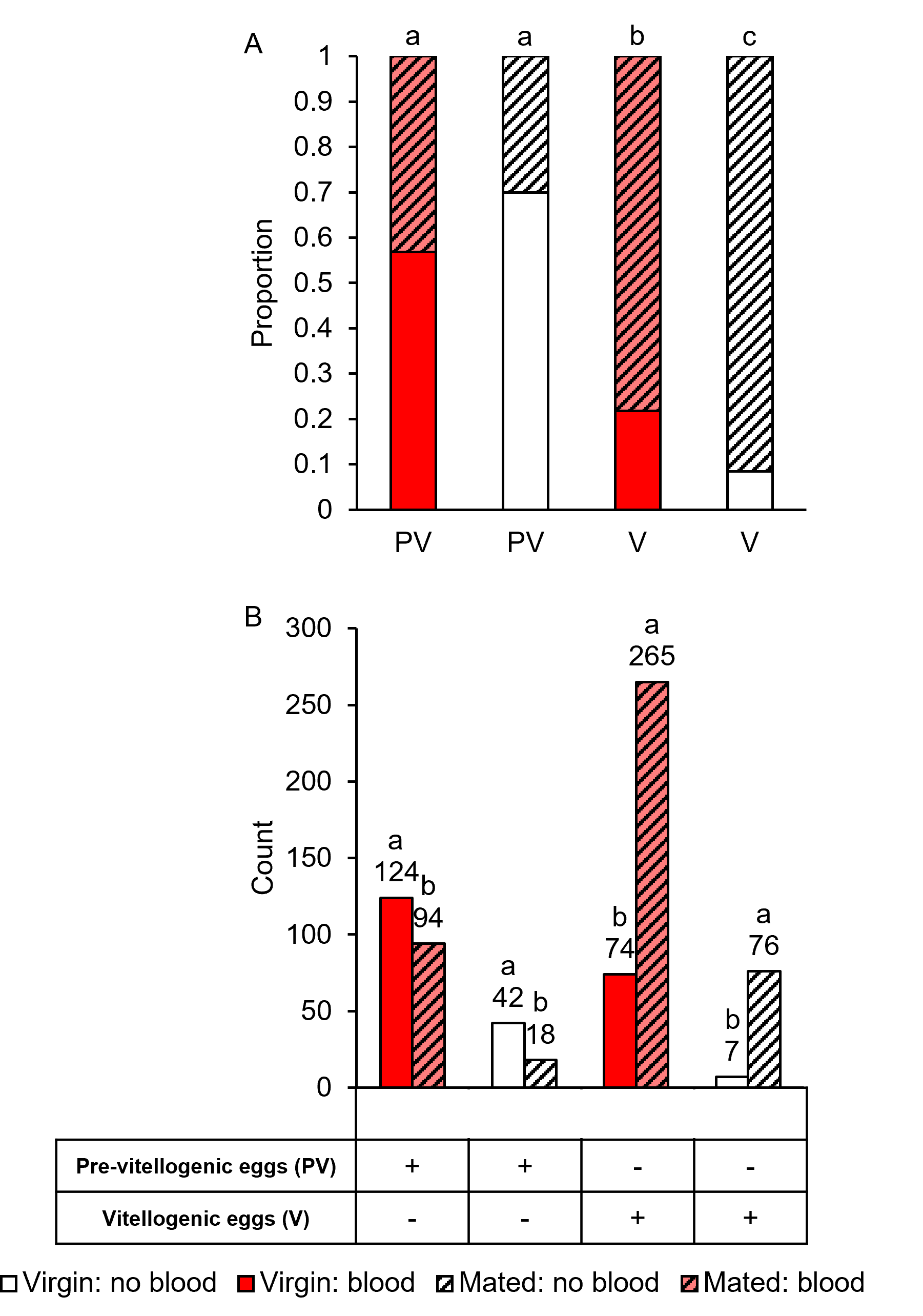

Supplement: S6 Fig — (A) Proportions of virgin and mated females differed significantly between combined blood and egg categories (Pearson chi-squared test: p<0.0001). (B) A disproportionate number of blood-fed and non-blood-fed virgins compared to mated females contained pre-vitellogenic eggs (BPV and NBPV, respectively) at the time of collection, indicating that they had likely not blood fed previously and had not yet completed a gonotrophic cycle. A disproportionate number of blood-fed and non-blood-fed mated females compared to virgins contained vitellogenic eggs (BV and NBV, respectively) at the time of collection, suggesting that many had likely blood fed previously and had at least partially undergone a prior gonotrophic cycle. Abbreviations: see far-left table column in B. Numbers above columns in B represent sample sizes. Letters above columns denote Bonferroni-corrected post-hoc comparison p-values. Comparisons in (A) are between stacked columns and comparisons in (B) are within categories. (TIF) [file pntd.0009815.s007.tif]

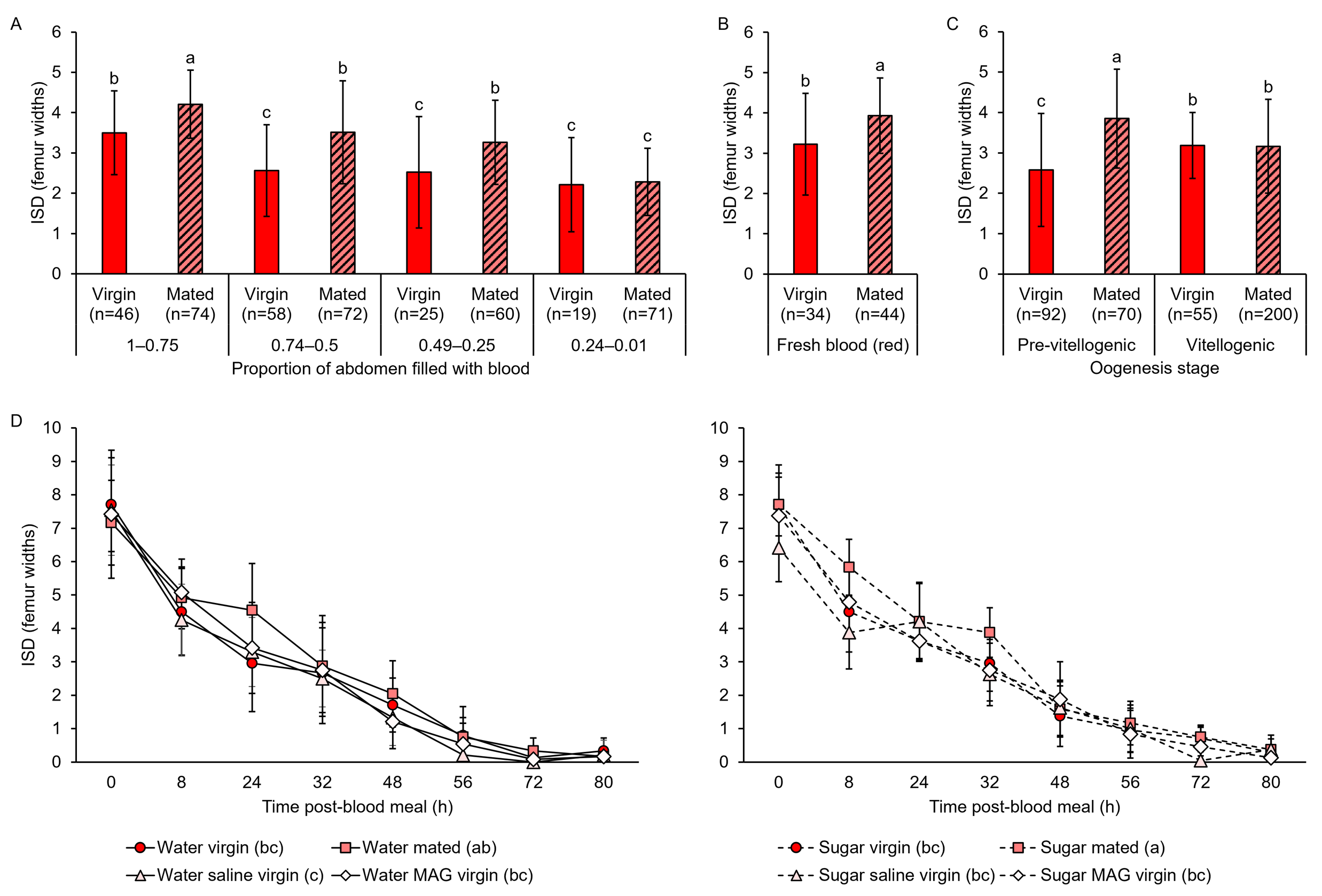

Supplement: S7 Fig — (A–C) For field-derived mosquitoes, female spermathecae were dissected to assess mating status. Mated field-collected mosquitoes from Medellín, Colombia displayed wider abdomens compared to virgins (A), even shortly after a blood meal as indicated by the presence of fresh blood (B). Virgin and mated females with pre-vitellogenic stage eggs differed in degree of abdominal distension, whereas virgin and mated females with vitellogenic stage eggs displayed similar degrees of distension (C). Laboratory experiments involving nulliparous Thai mosquitoes that had not previously blood fed (D) show that abdominal distension after an initial blood meal is not affected by mating or MAG injection (n = 96 total females per group). Error bars denote SD. Letters above columns in A and C and in parentheses next to the treatment group names in D denote H-B-corrected post-hoc comparison p-values and letters above columns in B denote independent samples T-test comparison p-values. (TIF) [file pntd.0009815.s008.tif]

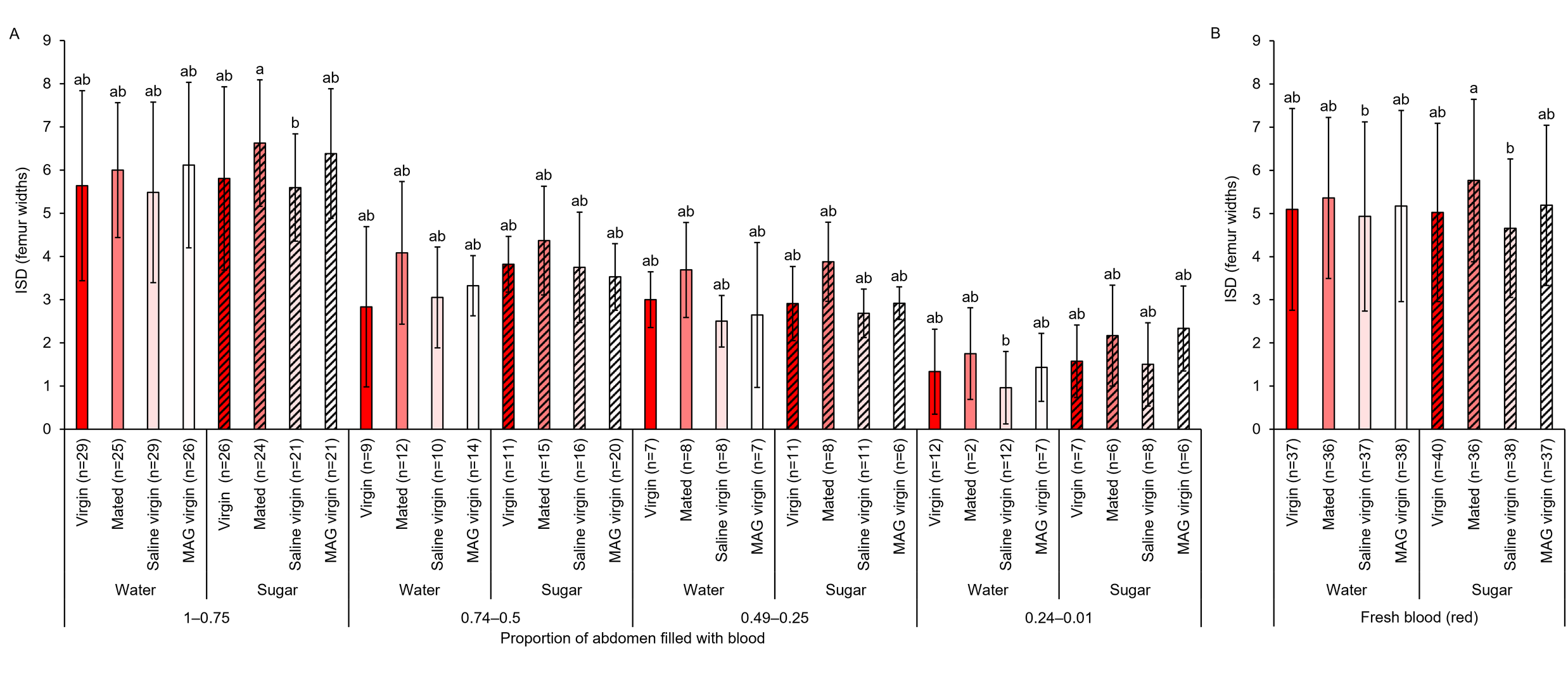

Supplement: S8 Fig — (A, B) Laboratory experiments involving nulliparous Thai mosquitoes that had not previously blood fed show that abdominal distension is generally similar between combined mating treatment and sugar feeding treatment groups in an initial blood meal. ISDs were similar whether compared by the proportion of the abdomen that was filled with blood (A) or by abdomens with fresh blood (B). Error bars denote SD. Letters above columns denote H-B post-hoc test comparison p-values. (TIF) [file pntd.0009815.s009.tif]
