# Supplementary material for: The impact of mating and sugar feeding on blood-feeding physiology and behavior in the arbovirus vector mosquito Aedes aegypti
Source: PLoS Negl Trop Dis. 2021 Sep 30;15(9):e0009815. doi: 10.1371/journal.pntd.0009815 (PMC8509887; doi:10.1371/journal.pntd.0009815)
Supplement: Attachment — Submitted filename: League et al._Responses to reviewer comments_PLOS NTDs.docx [file pntd.0009815.s013.docx]

| **Comment** | **Response** |
| --- | --- |
| **Reviewer #1** | |
| 1. Page 22, Fig. 3 versus Page 25 Fig. 5. The former presents data supporting that sugar but not mating or MAG affect avidity for an initial blood meal while the latter presents data that mating and MAG do affect avidity over successive meals. What causes some pause is the seemingly large differences in the proportion of females that initially fed between the two figures with 50-60% of virgin and mated females and 20-30% of saline and MAG females feeding in Fig. 3 while more than 90% of virgin and mated females and >75% of saline and MAG females fed at BM1 in Fig. 5. I recognize blood feeding is often variable with mosquitoes no matter how hard one tries to standardize conditions. But the data presented also makes it difficult to fully buy into concluding that mating and MAG affected outcomes over successive blood feeding opportunities when Fig. 3 shows responses that are so much lower than for BM1 in Fig. 5 but are fairly similar for the MAG-water/sugar treatment for BM2. Some comment/discussion about variation is thus potentially warranted in regard to the outcomes presented for blood-feeding avidity. | While the feeding timeline for Fig 3 and Fig 5 are identical (i.e., females were fed “on the first day post-mating treatment and sugar feeding treatment” at “2–3 days PE”) and host blood meal sources were presented for 5 min in each experiment, the experiments were designed to examine different aspects of feeding avidity and thus used different feeding arrangements.  The experiments in Fig 3 were primarily designed to measure latency to feeding and tested this metric by treatment group with 50 individuals fed simultaneously in the same container. Because of this, we used a large 6.5 L beaker with black cloth resting pads. Although this arrangement normalized starting position relative to the host arm and permitted measurements of latency on a large number of mosquitoes simultaneously while permitting video recording, it did lower the total number of mosquitoes that fed compared to other experiments because of the size and transparency of the container and the competing visual/tactile attractants of the black cloth.  Fig 5, by contrast, presented the blood meal source to 25 females per group with females held individually in much smaller, 0.5 L cups lacking any other competing sensory attractants. This made sense since the goal of this experiment was to measure feeding avidity in blood meals after an initial feeding, so the experiment was designed to give all females maximal time for each feeding, including the initial feeding, and then track what we anticipated would be more substantial differences in subsequent feedings.  To clarify these points, we added the following sentences to the revised manuscript:  Lines 222–224: “Feeding was permitted for 5 min under these conditions because for females at this age range, five min was sufficient to obtain a nearly saturating (≈85–100%) feeding avidity for each treatment group (see Fig 1C).”  Lines 287–291: “Although resting pads offered competing visual and tactile attractants, and thus contributed to lower feeding avidity (≈20–60%) compared to similar experiments (see Figs 1, 4, and 5), they were effective in standardizing mosquito resting position prior to host presentation in the larger, transparent holding containers used in these experiments (see Fig 3A).”  Lines 310–312: “Feeding was permitted for two min, rather than for five min, to obtain potentially discriminating, but non-saturating feeding avidities (i.e., ≈60–70%) in these experiments, which featured greater host skin surface area for feeding compared to the other feeding avidity experiments.”  Lines 322–324: “Feeding was permitted for five min in these experiments because this provided ample opportunity for most individually held mosquitoes of this age to obtain an initial feeding (≈80–100%) as well as a subsequent feeding.” |
| 2. The Discussion section of the manuscript talks about mating reducing blood-feeding avidity over successive meals being linked to egg development and egg laying, which the authors present some results for in Fig. S4. My suggestion is that the authors elaborate a bit more on what they present in S4 and write on page 31. Specifically, several studies in the literature indicate that blood feeding stimulates egg maturation (vitellogenesis) in both virgin and mated females but virgin females very much differ from mated females in regard to oviposition with the latter usually laying many eggs in clutch and the former laying few or no eggs. Judson (1967) that the authors cite in the manuscript further report that implantation of accessory glands stimulated virgins to oviposit the same number of eggs as mated females. In contrast, I do not know if the MAG prep the authors inject similarly stimulates virgins to oviposit comparable numbers of eggs as mated females because Fig. S4 doesn’t show this and I haven’t seen results elsewhere that clearly indicates MAG preps yield the same outcome as accessory gland implantation did in Judson’s assays. So, this point I think needs to be addressed in the manuscript. The other small suggestion, if MAG injection causes virgins to oviposit similar numbers of eggs, is that the data in Fig. 5 seems to suggest mating/MAG -induced declines in feeding avidity are tied more to egg laying than egg development since virgin females that blood feed produce as many mature eggs as mated females. If MAG injection does not stimulate virgins to lay a similar number of eggs as virgins, then something other than oviposition is at play to yield the blood feeding patterns shown in Fig. 5. | Although our method of injecting male accessory gland lysate differed from Judson’s injection of an entire isolated male accessory gland, his finding that “Implantation of male accessory gland into virgin females causes them to oviposit on maturation of their eggs” was confirmed in what is now S5 Fig, where we note that “Mated and MAG-treated females reach their peak egg laying activity between the second and third as well as the fourth and fifth blood meals.” The graphs show that in both water- and sugar-treated groups, mated and MAG treatments yielded identical proportions of ovipositing females in each group.  The reviewer is correct that unlike Judson we did not count the number of eggs laid per female in this experiment; we only noted whether the females had laid eggs at each blood feeding (which Judson also did). This was because tracking oviposition was not the aim of this experiment and similar experiments have already been conducted across single and multiple blood meals in our lab (Villarreal et al., 2018). Please note that in that study, which examined the same Thai mosquito strains used in our present work, non-injected virgin and saline-injected virgin females produced significantly fewer eggs in addition to laying fewer eggs compared to MAG-injected and mated females. This result contrasts with Judson’s study, which examined these phenomena in the Liverpool *Ae. aegypti* strain. In our experience, Thai *Ae. aegypti* strains show a great deal of variation in egg production in response to mating/MAG fluids and highly lab-adapted strains usually show fewer effects. For this reason, we utilize a field-relevant Thai strain that is supplemented with wild caught mosquitoes regularly.  Although non-injected virgins and saline-injected virgins do not produce as many eggs as mated females and MAG-injected virgins in our strain, we agree that the declines in feeding avidity that we observed were primarily due to oviposition. We clarified this, and the above points, in lines 653–664 of the revised manuscript:  “Seminal fluid proteins allow females to develop, fertilize, and lay eggs [16–26], and our data shows that mated and MAG-injected females experience similar declines in feeding avidity during periods of equivalent egg laying activity (Fig 5; S5 Fig). Previous work from our lab on the same Thai strain studied here showed that egg production and laying is similarly elevated in mated females and MAG-injected females compared to non-injected virgins and saline-injected virgins [40]. Although work by Judson in *Ae. aegypti* showed that blood feeding stimulates equivalent egg production (though not egg laying) in virgin and mated or MAG-treated females [19], this difference likely stems from that study’s use of the highly lab-adapted Liverpool strain compared to our studies of a field-relevant Thai strain which is regularly supplemented with wild-caught mosquitoes. Taken together, we conclude that oviposition is a major determinant of *Ae. aegypti* host feeding patterns across multiple blood meals. The feeding avidity patterns that we observed may also reflect […].” |
| 3. Fig. 6 and S5. The pie charts don’t seem essential in this figure as a reader can glean the numbers of individuals in each outcome from the N values at the bottom of the bar graphs. If the pie charts were eliminated, the authors could also perhaps make Fig. 6A, B with B showing what is currently presented in S5 (thus eliminating S5). In this way, it would be easier on a reader to see how presence of blood and vitellogenic eggs in the ovaries relate to one another. Maybe I don’t fully understand what’s written on page 26 but it would seem to me difficult to know from the data presented that the mated females were actually collected at different ages/or stages of their reproductive life histories (or had more likely blood fed previously-line 732) based on what is presented in Fig. 6 and S5. If the authors are confident this is the case, rewording is needed to clarify the reasoning why the data presented support this. | We agree that Fig 6A and S5A are redundant and have removed these panels. We also moved Fig S5B into Fig 6 as Fig 6B to allow for easier comparison between these data and adjusted the figure legend accordingly (see lines 594–595).  Regarding our statements on p. 26 of the original manuscript, we have modified and/or qualified our claims in lines 572–573 and 580–589 to better reflect the nuances of these data:  “**Mating status does not affect the likelihood of containing a blood meal in a natural disease endemic field setting.**”  “Because females that are mated or contain vitellogenic stage eggs would on average be older than females that are virgin or do not contain mature eggs, the females we collected may have differed by age and/or gonotrophic cycle stage. These observations show that, in natural urban field settings, virgin and mated females are equally likely to contain blood, but mated females tended to be in more advanced stages of the gonotrophic cycle compared to virgins (for further details, see S6 Fig). Although this may also suggest that mated females (particularly those with substantial amounts of undigested blood and fully developed eggs) are more likely than virgins to have ingested multiple blood meals, we cannot rule out the possibility that females that contained small amounts of blood developed eggs from their current blood meal.” |
| 4. Fig. 7. I appreciate that the authors had hoped abdomen distention could be used a proxy for assessing blood feeding amounts, but the confounding effects of egg development and other factors make the outcomes of this part of the manuscript uncertain and also raise questions about using this measure for assessing blood meal sizes in the field. So, it may be more appropriate to shorten this section and move Fig. 7 to the Supp. Files. | We agree and have moved Fig 7 (Now S7 Fig) and its accompanying Results text (now S1 Text) to the Supporting information. We have added a brief comment on this data in lines 597–603 of the main text Results:  Finally, although measurements of blood meal engorgement in field-collected Colombian mosquitoes suggest that mated females display greater abdominal distension compared to virgins, this was not the case in Thai mosquitoes examined in the laboratory (for details, see S1 Text and S7 and S8 Figs). Together, our results indicate that under controlled conditions where egg development and other factors are accounted for, mating and MAG do not necessarily lead to increased blood meal engorgement in an initial blood meal. However, additional work is needed to understand their effects in the field. |
| 5. Line 688. I am familiar with refs 103 and 104. I agree sugar and blood feeding can both meet energetic/maintenance demands but I respectfully disagree if the authors are suggesting sugar and blood feeding can meet similar demands when it comes to egg production as I’ve never seen that sugar feeding stimulates vitellogenesis or oviposition to anything remotely comparable to blood feeding in aegypti or other anautogenous species. | We completely agree and we see how our original wording could lead to this misconception. Accordingly, we modified lines 699–701 as follows:  “Although blood, rather than sugar, is required for egg production in anautogenous species, these observations make sense considering that both sugar and blood feeding can meet other energetic demands [104,105].” |
| 6. The authors may want to consider presenting their vectorial capacity estimates as part of the Results rather as a point of Discussion given the methods used for the calculations presented are presented in the Methods section and rely on data generated in earlier parts of the study. Line 755, the word across looks like it should be deleted. | We agree with this recommendation. In addition to discussing these points in lines 761–781 of the Discussion, we have moved the vectorial capacity estimates from the Discission to the Results, adding the following text to lines 604–610:  “**Mating, MAG extract, and sugar feeding could impact Aedes vectorial capacity estimates.**  We used feeding avidity data from both our Thai laboratory and Colombia field strain(s) to calculate human-biting rates for each of our mating and sugar feeding treatment groups to determine the effect these factors might ultimately have on vectorial capacity (Table 2). These estimates show that mating and sugar feeding reduce females’ biting rates, and hence may reduce vectorial capacity estimates. Thus, non-sugar fed virgin females may transmit arboviruses at higher rates relative to mated females that have fed on sugar.”  Further, we corrected an error in our calculations that led to different numerical values; the trends we see upon recalculation are identical to what we originally reported and the manuscript has been modified accordingly (see lines 780–781).  We also deleted the word “across” from line 755 (now line 770) and thank the reviewer for catching this typo. Lastly, |
| **Reviewer #2** | |
| 1. These findings clarify longstanding conflicting results and are relevant for researchers studying many components of mosquito behavior and physiology, however some of the key findings are obscured by the current presentation of the data (for example water vs sugar fed differences compared directly). | We address this general comment on improvements to the presentation of key findings (including the one alluded to in this comment) in the specific comments below. |
| 2. The calculations for vectorial capacity were not well integrated into the paper. This could either be removed or tied to the findings or predicted outcomes to more directly address public health relevance. | We agree with this recommendation. In addition to discussing these points in lines 761–781 of the Discussion, we have moved the vectorial capacity estimates from the Discission to the Results, adding the following text to lines 604–610:  “**Mating, MAG extract, and sugar feeding could impact Aedes vectorial capacity estimates.**  We used feeding avidity data from both our Thai laboratory and Colombia field strain(s) to calculate human-biting rates for each of our mating and sugar feeding treatment groups to determine the effect these factors might ultimately have on vectorial capacity (Table 2). These estimates show that mating and sugar feeding reduce females’ biting rates, and hence may reduce vectorial capacity estimates. Thus, non-sugar fed virgin females may transmit arboviruses at higher rates relative to mated females that have fed on sugar.”  Further, we corrected an error in our calculations that led to different numerical values; the trends we see upon recalculation are identical to what we originally reported and the manuscript has been modified accordingly (see lines 780–781). |
| 3. Some of the claims (for example, in Figure S3 that variability in sugar consumption leads to variability in host-seeking) could be strengthened by quantification of sugar consumption and the major findings could be more clearly communicated through modifications to the figures and text. | We agree that the data presented in S3 Fig would be strengthened by quantifying individual sugar ingestion prior to blood feeding and noted this in the Results and at length in the Discussion (lines 497–499 and 688–695 of the original manuscript, respectively). Given that the trend in S3 Fig across individual trials did not come through in the combined data analysis presented in Fig 4, we have removed the phrase “, but sugar feeding does” from the figure legend in line 537. Further, although not included in the manuscript due to their preliminary nature, we conducted pilot studies using sugar-water dyed with blue food coloring that indicated widespread and uniform sugar meal ingestion prior to blood feeding. Thus, while we agree that this aspect of the experiment could be further investigated in follow-up studies, we believe it does not impact our main findings, as all sugar groups were provided equal access to sugar. That said, to better emphasize this point, we have further qualified our presentation of this data in the Results (lines 532–535):  “Taken together, these results show that in an initial blood meal, mating and MAG do not impact female feeding avidity. However due to apparent differences in sugar feeding that require further investigation, consumption of sugar, to the extent that it occurs, may impact a female’s tendency to blood feed.”  And in the Discussion (lines 706–710):  “Whatever the precise causes, these observations merit further study, including by developing a means of quantifying individual sugar consumption prior to blood feeding that does not involve destruction of the mosquito in the process. Together, our findings suggest that future studies examining blood feeding should account for prior sugar feeding as an important potential source of variation.” |
| 4. Lines 84 – 87 – “In Ae. aegypti, the transfer of male accessory gland fluid (i.e., seminal fluid) proteins to females during mating renders females resistant to subsequent male mating attempts [8–15], increases egg development and oviposition [16,17,26,18–25], inhibits courtship acoustic harmonization [27], decreases flight activity [28–33], reduces host seeking behavior [18,34–39], increases survival [40], and induces immune responses [41,42]. | We thank the reviewer for noting these differences in the verbs in this sentence and added an “s” to the end of all the verbs in this list to agree with the first verb “renders” (see lines 82–86). |
| 5. Figure 2c and d – Should this y axis read mg (instead of g)? | We thank the reviewer for noting this mistake and made this correction to the Y-axis of what is now Fig 2B. |
| 6. Figure 4a – There is a group referred to as “sugar water fed” and this is the only instance of this usage (presumably to refer to “sugar fed”). | While sugar water treatment groups are identified as simply “Sugar” treated in the Methods, we thank the reviewer for noting this inconsistency and made the suggested change to the label in Figure 4a. |
| 7. Table 1 – Typo: Seaton and Lumsden Timing of feeding PE should be 3 – 4 d PE | We thank the reviewer for pointing out this omission and have made this correction to Table 1. |
| 8. Lines 98 – It would be helpful to mention here that blood feeding and mating can happen in either order for successful reproduction and that virgins are capable of developing eggs and oviposition (as shown in Figure S4). | We agree that mentioning mating and blood feeding order would be a helpful point to make at this juncture, and have modified the text of lines 96–98 as follows:  “As females that approach hosts to blood feed are often intercepted and inseminated by swarming males either before or after obtaining a blood meal [49], researchers have long suspected potential biological crosstalk between mating and blood feeding.  We now address the second point regarding virgins developing and even laying some eggs in lines 653–664 of the revised manuscript:  “Seminal fluid proteins allow females to develop, fertilize, and lay eggs [16–26], and our data shows that mated and MAG-injected females experience similar declines in feeding avidity during periods of equivalent egg laying activity (Fig 5; S5 Fig). Previous work from our lab on the same Thai strain studied here showed that egg production and laying is similarly elevated in mated females and MAG-injected females compared to non-injected virgins and saline-injected virgins [40]. Although work by Judson in *Ae. aegypti* showed that blood feeding stimulates equivalent egg production (though not egg laying) in virgin and mated or MAG-treated females [19], this difference likely stems from that study’s use of the highly lab-adapted Liverpool strain compared to our findings in a field-relevant Thai strain which is regularly supplemented with wild-caught mosquitoes. Taken together, we conclude that oviposition is a major determinant of *Ae. aegypti* host feeding patterns across multiple blood meals. The feeding avidity patterns that we observed may also reflect […].” |
| 9. Line 143 – A more detailed explanation of “water-fed controls” would be helpful here. Does this refer to females who were provided exclusively water as adults with no access to sugar? | We added a clarification on this point to lines 143–144 as follows:  “However, these trends were inconsistent across trials, feedings took place at room temperature, and the experiments did not include water-fed controls (i.e., water alone, with no sugar added), with females provided continual access to sugar except for the day before each blood feeding.” |
| 10. Line 199 – 202 Why was the male:female ratio changed between these experiments? Also it’s not clear if males were removed for the 2-3 day period when mosquitoes were held prior to feeding. | For the blood meal size and digestion experiments, males and females in the mated group were held together for two days, rather than only one day as was the case for the feeding avidity experiments. We found that a 2:1 male:female ratio in the avidity experiments ensured that all females mated prior to blood feeding in this shortened holding period (for more details, see responses to comments 11 and 12 below). To clarify when males were removed from mated group containers, we made the following changes to lines 211–213:  “After these mating periods, males were removed from mated group buckets prior to blood feeding to allow for female recovery and feeding in the absence of male harassment. Mosquitoes were held for a total of one to three days […].” |
| 11. Line 208 – A graphical timeline of the protocols starting with mosquito eclosion would help the reader visualize the order and timing of injection/mating and feeding – and this would be especially helpful in comparing the blood meal size vs avidity experiments. | We have included a graphical treatment timeline (S2 Fig), which is now mentioned in lines 214–215 of the revised manuscript:  “[…] (see S2 Fig for a graphical treatment timeline).” |
| 12. Line 215 – Was mating status verified for females used in the meal size and digestion experiments? | In preliminary experiments using the same treatment groups under identical experimental mating conditions, male:female ratios, and mating time periods for each set of experiments performed in our study, we verified that 100% of mated group females contained sperm in their spermathecae. Because of this, and other experimental considerations (e.g., the need to preserve female abdomens for hemoglobin measurements, which involved homogenization of entire intact specimens), we performed spermathecal dissections (e.g., verification of mating status) in some, but not all, experiments. However, our pilot data from these experiments and other experiments in the lab consistently show that groups of Thai strain females are fully mated at the male:female ratios we used over a period of only 1–2 hours, so our holding times of one to two days are extremely conservative and have always yielded 100% female mating rates in prior work in our lab (see Villarreal et al., 2018, etc.). These points are summarized in lines 205–211 of the revised manuscript:  “Sex ratios and holding times were based on previous work in our lab [40] and pilot experiments confirmed that they yielded 100% female-mating rates prior to blood feeding. For these reasons, as well as other experimental considerations (e.g., the need to preserve female abdomens for hemoglobin measurements; see below), we did not perform spermathecal dissections to verify mating status after blood meal size and blood meal digestion experiments, or in some of our feeding avidity experiments (initial and multiple blood meal experiments on human host forearm).” |
| 13. Line 268 - 270 The authors choose to test 2 - 3 days PE timepoint for feeding to mimic more naturalistic access to hosts. These feeding rates in the experiments shown in Figure 3 are lower compared to those in their other experiments – could this be due to immature females or is this related to assay design? | As noted in lines 276–278, females are capable of blood feeding between 12–24 h post-eclosion at our holding temperatures, so our 2–3 days post-eclosion feeding time point permitted more than enough time for full female maturity with respect to reproduction and host-feeding. Differences in the proportion of females that blood fed between experiments were largely due to differences in experimental design. While the feeding timeline for Fig 3 and Fig 5 are identical (i.e., females were fed “on the first day post-mating treatment and sugar feeding treatment” at “2–3 days PE”) and host blood meal sources were presented for 5 min in each experiment, the experiments were designed to examine different aspects of feeding avidity and thus used different feeding arrangements.  The experiments in Fig 3 were primarily designed to measure latency to feeding and tested this metric by treatment group with 50 individuals fed simultaneously in the same container. Because of this, we used a large 6.5 L beaker with black cloth resting pads. Although this arrangement normalized starting position relative to the host arm and permitted measurements of latency on a large number of mosquitoes simultaneously while permitting video recording, it did lower the total number of mosquitoes that fed compared to other experiments because of the size and transparency of the container and the competing visual/tactile attractants of the black cloth  Fig 5, by contrast, presented the blood meal source to 25 females per group with females held individually in much smaller, 0.5 L cups lacking any other competing sensory attractants. This made sense since the goal of this experiment was to measure feeding avidity in blood meals after an initial feeding, so the experiment was designed to give all females maximal time for each feeding, including the initial feeding, and then track what we anticipated would be more substantial differences in subsequent feedings.  To clarify these points, we added the following points to the revised manuscript:  Lines 222–224: “Feeding was permitted for 5 min under these conditions because for females at this age range, five min was sufficient to obtain a nearly saturating (≈85–100%) feeding avidity for each treatment group (see Fig 1C).”  Lines 287–291: “Although resting pads offered competing visual and tactile attractants, and thus contributed to lower feeding avidity (≈20–60%) compared to similar experiments (see Figs 1, 4, and 5), they were effective in standardizing mosquito resting position prior to host presentation in the larger, transparent holding containers used in these experiments (see Fig 3A).”  Lines 310–312: “Feeding was permitted for two min, rather than for five min, to obtain potentially discriminating, but non-saturating feeding avidities (i.e., ≈60–70%) in these experiments, which featured greater host skin surface area for feeding compared to the other feeding avidity experiments.”  Lines 322–324: “Feeding was permitted for five min in these experiments because this provided ample opportunity for most individually held mosquitoes of this age to obtain an initial feeding (≈80–100%) as well as a subsequent feeding.” |
| 14. Line 279 - 281 – The authors state that “Video analyses were performed using Adobe Premiere Pro 2020 (Adobe Inc., San Jose, CA, USA) to calculate the latency to blood feeding, defined as the time that elapsed from host presentation to the initiation of feeding for each individual female.” but it’s not clear that they can distinguish individual females to be sure that each female only fed one time during the assay? | Since the host blood meal source in these experiments was a stationary forearm, the vast majority of females that blood fed did so in the same location on the forearm that they initially landed on for the duration of their feeding. Typically, females left only after detaching and dropping off the host once they had fed to repletion. In the rare event that a female moved to a nearby location on the arm due to overcrowding, the female was fully visible and trackable. Further, the assay only took into account latency to initial feeding, so even if a female moved to different section of the arm, this second phase of feeding would not be counted and the female would be detectable as already having started a meal due to visible blood engorgement in her abdomen. These points have now been incorporated into lines 299–303 of the Methods:  “Since the forearm was stationary for the duration of the assay, females that blood fed nearly always did so at same location on the host on which they initially landed. In the rare event when a female moved to reinitiate feeding at a nearby location on the host, the partially engorged female was fully trackable and the subsequent phase of feeding was excluded from latency measures.” |
| 15. Line 281 – How was “feeding initiation” scored? Was this landing on the forearm, skin penetration, or presence of blood/abdominal distension? | We defined feeding initiation as the first instance that a female landed on the host as probing, penetration of the skin, and ingestion of blood commenced immediately after and sometimes nearly simultaneous with landing (in the case of probing). Further, initial contact with the host was directly observable and unambiguous in our video recording set up. We have clarified our criterion for “feeding initiation” in lines 298–299 as follows:  “Feeding initiation was defined as the moment when a female first landed on the host forearm, after which probing and ingestion of blood immediately commenced.” |
| 16. Line 295 – What was the rationale for choosing 48 hours for refeeding timepoints? | We now make clear the rationale for 48 h blood feeding cycles in lines 324–326 of the revised Methods:  “Females were fed every 48 h, as this is the time interval at which most females feed in nature during a typical gonotrophic cycle [83] as well as the time required for complete digestion of a blood meal (see Results).” |
| 17. Figure 2 – Since sugar feeding seems to have the largest effect, it would be more intuitive to see direct comparisons of between water vs sugar (2a vs 2b) and to show direct comparisons between the relevant metrics for digestion/clearance rates. | The text of the Methods and Results note that water and sugar treatment groups were directly compared in all statistical analyses. This is also indicated by the H-B-corrected post-hoc comparison p-values included in Fig 2 (see letters in parentheses). We initially presented the data in Fig 2 as two graphs that combined panels A and B, as well as C and D. However, we felt that having data markers, error bars, and trend lines for eight treatment groups in the same graph led to overcrowded data than would not be easily discernible for readers. By contrast, we believe that separating the data by water/sugar treatment group allows for the salient trends to emerge between comparisons more clearly. However, to avoid the misimpression that these data were not directly compared, we condensed these figure panels into simply A (top two graphs) and B (bottom two graphs) panels with only a single Y-axis label for each pair of graphs. We also made the same changes to Fig 5 and S4 Fig. |
| 18. Lines 427 - 433 – There appear to be conflicting statements in the legend vs title of this figure: that sugar feeding slows digestion rates using hemoglobin measurements but that sugar feeding does not affect digestion rates according to mass measurements. Is it correct that the hemoglobin assay but not the weights support the title of this figure? | Although the title summarizes the overall trends, the reviewer is correct: mating and MAG injection did not alter blood digestion in either the hemoglobin or weight assays, but sugar feeding impacted blood digestion rates in only the hemoglobin assay (although masses were initially higher in the water-fed groups compared to sugar-fed groups, digestion rates as tracked by changes in mass were the same). Since the results summary in the Fig title lacks this nuance, we modified the title as follows (lines 462–463):  “**Fig 2. Mating and MAG injection do not alter blood meal digestion in an initial feeding, but sugar feeding slows hemoglobin digestion.**” |
| 19. Figure 5 – Adding time to the x axis would help to clarify these results. It would also be helpful to directly visualize water vs sugar fed. | We agree, and added day indicators into the X-axis of Fig 5 to match the labels in S5 Fig. As for directly visualizing water vs sugar fed groups in Fig 5, this was addressed in the response to comment 17 above:  The text of the Methods and Results note that water and sugar treatment groups were directly compared in all statistical analyses. This is also indicated by the H-B-corrected post-hoc comparison p-values included in Fig 2 (see letters in parentheses). We initially presented the data in Fig 2 as two graphs that combined panels A and B, as well as C and D. However, we felt that having data markers, error bars, and trend lines for eight treatment groups in the same graph led to overcrowded data than would not be easily discernible for readers. By contrast, we believe that separating the data by water/sugar treatment group allows for the salient trends to emerge between comparisons more clearly. However, to avoid the misimpression that these data were not directly compared, we condensed these figure panels into simply A (top two graphs) and B (bottom two graphs) panels with only a single Y-axis label for each pair of graphs. We also made the same changes to Fig 5 and S4 Fig. |
| 20. Figure 5 – Were females given constant access to egg laying substrate throughout this assay? | Females were provided constant access to egg laying substrate (i.e., water-soaked cotton pad) throughout the assay. We clarified this in greater detail in lines 326–327 of the Methods:  “For all trials, females were provided with constant access to a water-soaked cotton pad on a mesh cup lid for hydration and egg laying.” |
| 21. Figure 7a – It would be helpful to see some pictures of these measurements. | We added example measurements (white bars on abdomen and midleg femur) to Fig 1B to illustrate inter-sclerite distance measurements. This is now noted in the Fig 1 legend (lines 434–435):  “White bars in panel B illustrate inter-sclerite distance and femur width measurements used for blood meal engorgement estimates presented in S7 and S8 Figs.”  As well as in lines 267–269 of the Methods:  “Each female’s inter-sclerite distance, defined as the widest point of separation between the ventral sternites and the dorsal tergites, and midleg femur width was measured (see Fig 1B).” |
| 22. Figure S3 – Quantification of sugar consumption among the sugar-fed groups would be helpful to contextualize the variation in blood-feeding avidity here. It would be helpful to give some quantification of the range of meal sizes/calories consumed. | We address this in response to comment 3 above:  We agree that the data presented in S3 Fig would be strengthened by quantifying individual sugar ingestion prior to blood feeding and noted this in the Results and at length in the Discussion (lines 497–499 and 688–695 of the original manuscript, respectively). Given that the trend in S3 Fig across individual trials did not come across in the combined data analysis presented in Fig 4, we have removed the phrase “, but sugar feeding does” from the figure legend in line 537. Further, although not included in the manuscript due to their preliminary nature, we conducted pilot studies using sugar-water dyed with blue food coloring that indicated widespread and uniform sugar meal ingestion prior to blood feeding. Thus, while we agree that this aspect of the experiment could be further investigated in follow-up studies, we believe it does not impact our main findings, as all sugar groups were provided equal access to sugar. That said, to better emphasize this point, we have further qualified our presentation of this data in the Results (lines 533–535):  “However due to apparent differences in sugar feeding that require further investigation, consumption of sugar, to the extent that it occurs, may impact a female’s tendency to blood feed.”  And in the Discussion (lines 706–710):  “Whatever the precise causes, these observations merit further study, including by developing a means of quantifying individual sugar consumption prior to blood feeding that does not involve destruction of the mosquito in the process. Together, our findings suggest that future studies examining blood feeding should account for prior sugar feeding as an important potential source of variation.” |
| 23. Table S3 – The calculations for vectorial capacity are not well-integrated into the text. I would find more discussion of the predicted differences between the Thai field and Colombian strains to be useful or a prediction of outcomes for disease transmission in these sites. | We address this in response to comment 2 above:  We agree with this recommendation. In addition to discussing these points in lines 761–781 of the Discussion, we have moved the vectorial capacity estimates from the Discission to the Results, adding the following text to lines 604–610:  “**Mating, MAG extract, and sugar feeding could impact Aedes vectorial capacity estimates.**  We used feeding avidity data from both our Thai laboratory and Colombia field strain(s) to calculate human-biting rates for each of our mating and sugar feeding treatment groups to determine the effect these factors might ultimately have on vectorial capacity (Table 2). These estimates show that mating and sugar feeding reduce females’ biting rates, and hence may reduce vectorial capacity estimates. Thus, non-sugar fed virgin females may transmit arboviruses at higher rates relative to mated females that have fed on sugar.”  Further, we corrected an error in our calculations that led to different numerical values; the trends we see upon recalculation are identical to what we originally reported and the manuscript has been modified accordingly (see lines 780–781). |
| **Reviewer #3** | |
| 1. The methods are acceptable. The one specific comment is were the mosquitoes acquired from a single cage and how were the 96 analyzed, such as were these measured in smaller cohorts from multiple cages. Please clarify as we get odd cage effects and mosquitoes from the same cage could more of less be considered technical rather than biological replicates. | The Thai mosquito colony used for these experiments is supplemented annually with eggs from field-collected mosquitoes from Thailand to maintain natural levels of genetic heterogeneity. The 96 mosquitoes per group in our blood digestion experiments were the totals from 32 females per treatment group over three trials. Each trial originated from a separate generation of eggs produced by our Thai colony. To account for batch effects between trials, we included a trial main effect in all of our statistical models. Although most of these points already touched upon in the Methods, we added the following clarifications added to lines 170–175:  “This colony has been supplemented annually with F_1_ eggs since 2009 to maintain natural levels of genetic heterogeneity. Individual trials for each set of experiments represented true biological replicates that originated from a separate generation of eggs produced by the Thai colony. Adults arising from colony eggs were held in separate 8 L plastic bucket cages for each experimental trial to avoid cage effects.” |
| 2. The results are clear, but I would suggest moving the vectorial capacity section to the end of the results. | We agree with this recommendation. In addition to discussing these points in lines 761–781 of the Discussion, we have moved the vectorial capacity estimates from the Discission to the Results, adding the following text to lines 604–610:  “**Mating, MAG extract, and sugar feeding could impact Aedes vectorial capacity estimates.**  We used feeding avidity data from both our Thai laboratory and Colombia field strain(s) to calculate human-biting rates for each of our mating and sugar feeding treatment groups to determine the effect these factors might ultimately have on vectorial capacity (Table 2). These estimates show that mating and sugar feeding reduce females’ biting rates, and hence may reduce vectorial capacity estimates. Thus, non-sugar fed virgin females may transmit arboviruses at higher rates relative to mated females that have fed on sugar.”  Further, we corrected an error in our calculations that led to different numerical values; the trends we see upon recalculation are identical to what we originally reported and the manuscript has been modified accordingly (see lines 780–781). |
| 3. Also, the marking of statistical significance on the figures needs clarification. As an example, Figure 4 shows no differences, but sugar feeding is discussed as different. I believe this stems from using a basic letter system, rather that bars showing p values to highlight the specific comparisons. This would improve clarity. | The letters above the columns indicating statistical similarity between groups (all “a”) when compared using Holm-Bonferroni-corrected p-values agrees with the effect of sugar in this set of experiments as outlined in lines 527–529:  “In whole host feeding trials, combined mating treatment and sugar feeding treatment did not influence feeding avidity in an initial blood meal (GZLM: treatment, p=0.091; trial, p<0.001; H-B post-hoc tests; Fig 4B).”  We tried bars/p-values as suggested, but it added significantly to the clutter of the figures, some of which (e.g., S2 and S3 Figs) entail numerous statistically significant post-hoc comparisons. Given that many experiments had 16 treatment groups, we believe that using APA-style letter subscripts offers a simpler method for denoting significant differences between groups. We double checked and confirmed that the lettering lines up with the write-up in the text.  Although sugar feeding generally impacted blood feeding across our experiments, we discuss potential reasons for the lack of a consistent effect in the whole human host feeding avidity trials in greater detail in revised portions of the Results and Discussion (see below). We agree that the data presented in S3 Fig would be strengthened by quantifying individual sugar ingestion prior to blood feeding and noted this in the Results and at length in the Discussion (lines 497–499 and 688–695 of the original manuscript, respectively). Given that the trend in S3 Fig across individual trials did not come across in the combined data analysis presented in Fig 4, we have removed the phrase “, but sugar feeding does” from the figure legend in line 537. Further, although not included in the manuscript due to their preliminary nature, we conducted pilot studies using sugar-water dyed with blue food coloring that indicated widespread and uniform sugar meal ingestion prior to blood feeding. Thus, while we agree that this aspect of the experiment could be further investigated in follow-up studies, we believe it does not impact our main findings, as all sugar groups were provided equal access to sugar. That said, to better emphasize this point, we have further qualified our presentation of this data in the Results (lines 532–535):  “Taken together, these results show that in an initial blood meal, mating and MAG do not impact female feeding avidity. However due to apparent differences in sugar feeding that require further investigation, consumption of sugar, to the extent that it occurs, may impact a female’s tendency to blood feed.”  And in the Discussion (lines 706–710):  “Whatever the precise causes, these observations merit further study, including by developing a means of quantifying individual sugar consumption prior to blood feeding that does not involve destruction of the mosquito in the process. Together, our findings suggest that future studies examining blood feeding should account for prior sugar feeding as an important potential source of variation.” |
| 4. Conclusions are sufficient. I'd removed the references to figures and such in the discussion, as these are included in the results and having them repeated is not necessary. | The PLOS NTDs Submission Guidelines do not mention avoiding references to Results in the Discussion section (see Discussion section under “Parts of a Submission”). We make frequent and specific references to aspects of our data in the Discussion, and the manuscript contains 14 figures (including Supporting information). After considering this suggestion, we believe figure references will be helpful to our readers to pinpoint the data we are referring to in this section, and thus have elected to leave them in. |
